# Norepinephrine derepresses the Fur regulon of *Neisseria gonorrhoeae* to enable growth in iron-limited conditions

**DOI:** 10.1128/jb.00597-25

**Published:** 2026-01-20

**Authors:** Camille S. Westlake, Julie L. Stoudenmire, Indu Bhatia, Yasiru R. Perera, Rachel M. Krueger, Cynthia Nau Cornelissen, Walter J. Chazin, Melissa M. Kendall, Alison K. Criss

**Affiliations:** 1 Department of Microbiology, Immunology, and Cancer Biology, University of Virginia 174484, Charlottesville, Virginia, USA; 2 Institute for Biomedical Sciences, Georgia State University 439338, Atlanta, Georgia, USA; 3 Department of Biochemistry, Vanderbilt University 215875, Nashville, Tennessee, USA; 4 Center for Structural Biology, Vanderbilt University 5718, Nashville, Tennessee, USA; 5 Department of Chemistry, Vanderbilt University 5718, Nashville, Tennessee, USA; Dartmouth College Geisel School of Medicine, Hanover, New Hampshire, USA

**Keywords:** *Neisseria gonorrhoeae*, iron regulation, catecholamine, iron acquisition

## Abstract

*Neisseria gonorrhoeae* (Gc) is the gram-negative bacterium that causes gonorrhea, a prevalent sexually transmitted infection that can have life-threatening clinical sequelae. Gc requires iron for human infection and uses the iron-responsive, iron-binding transcriptional repressor Fur to maintain iron homeostasis. Gc infects mucosal sites, where the neuroendocrine hormone norepinephrine (NE) is produced by the autonomic nervous system and various epithelial and immune cell types. Here, we show that NE derepresses the Fur regulon to alter bacterial iron homeostasis and increase Gc survival. By RNA-seq, we determined that NE rewires gonococcal gene expression to increase capacity for iron uptake while enabling increased intracellular iron availability. Of the 30 genes that were differentially expressed in NE-treated compared to untreated bacteria, 27 have Fur box-containing promoters. A possible mechanism for how NE derepresses the Fur regulon is through direct demetalation of Fur, as NE directly decreased binding of Fur *in vitro* to a DNA amplicon containing the Fur-binding sequence from Gc *tbpB*. NE increased the labile intracellular iron pool in Gc, evidenced by increased streptonigrin sensitivity, without significantly increasing the total bacterial iron content, suggesting that NE leads to the redistribution of cellular iron. The work presented here provides a novel mechanism for how Gc survives iron limitation within its obligate human host.

**IMPORTANCE:**

*Neisseria gonorrhoeae* (Gc) is the bacterial pathogen that causes gonorrhea, a sexually transmitted infection with an estimated global annual incidence of 87 million individuals. During infection, Gc must overcome iron limitation imposed by nutritional immunity. Here, we show that the host neuroendocrine hormone norepinephrine, which is present at the mucosal surfaces Gc infects, promotes the survival of iron-limited Gc. Our results support a novel mechanism by which norepinephrine works through the ferric uptake regulator, Fur, to enhance the capacity of Gc to take up iron and make it bioavailable. Our findings show that Gc responds to host-derived cues that enable it to resist iron limitation.

## INTRODUCTION

Gonorrhea, the sexually transmitted infection caused by the gram-negative bacterium *Neisseria gonorrhoeae* (Gc), is an ongoing threat to global public health that impacts an estimated 87 million people each year (1, 2). Untreated infections can cause severe clinical sequelae, including life-threatening ectopic pregnancy, urogenital strictures, congenital infections, endocarditis, and meningitis (1–4). With rising drug resistance and no effective vaccine, new approaches to treat and prevent gonorrhea are a high priority (5). One such approach is to target the mechanisms required by Gc to survive in its obligate human host (6–8).

Gc infects the urogenital, anorectal, oropharyngeal, and conjunctival mucosa, where it stimulates the recruitment of neutrophils. The vasculature, smooth muscle, and epithelia of these mucosal tissues are innervated by the sympathetic nervous system, which facilitates vital physiological functions (9–13). The catecholamine norepinephrine (NE) is the primary neurotransmitter of the sympathetic nervous system; NE is also released into systemic circulation by the adrenal glands (14). There is a growing body of research demonstrating the importance of catecholamine release by epithelial cells and various immune cell types, including neutrophils (15–24).

NE and other catecholamines have been reported to enhance virulence of many bacterial pathogens in two ways. First, bacteria can recognize catecholamines to stimulate trans-kingdom signaling-dependent changes in gene expression (25–34). The best-described system is in enterohemorrhagic *Escherichia coli*, in which QseC and QseE two-component inner membrane receptors bind NE and epinephrine, resulting in virulence factor induction (25–28). Catecholamine sensing by QseC is blocked by phentolamine, a competitive antagonist of mammalian ⍺-adrenergic receptors, in *E. coli*, *Vibrio cholerae*, *Aggregatibacter actinomycetemcomitans*, and *Salmonella enterica* (25, 33–36). Second, catecholamines, including NE, can enable bacteria to overcome iron limitation by promoting the reduction from Fe^3+^ to Fe^2+^ in host Fe^3+^-binding proteins like transferrin (37–40). This results in the dissociation of protein-bound iron ions, which can be used by a wide range of bacterial pathogens including *Staphylococcus epidermidis*, *Listeria monocytogenes*, and *Pseudomonas aeruginosa* (41–43). It has also been proposed that catecholamines may act as pseudosiderophores to shuttle iron into some bacteria, as the effects of catecholamines are dependent on TonB and TonB-dependent siderophore transporters (40, 43–45). The effect of catecholamines on bacterial growth under iron-limiting conditions has been reported to be QseC dependent in *A. actinomycetemcomitans* and *S. enterica* (35, 36).

Most organisms, including Gc, require iron (46, 47). Host metalloproteins, including transferrin, lactoferrin, and hemoglobin, limit the iron availability to microbes and restrict pathogen replication across all physiological niches. During infection, extracellular iron concentrations decrease even further as iron is shuttled into host cells (48). Sequestration of iron and other nutrient metals from bacteria has been termed nutritional immunity (49). Gc overcomes nutritional immunity by expressing outer membrane TonB-dependent transporters (TdTs) that bind human metal-binding proteins and extract the metal from them (reviewed in references 50–52). The characterized TdTs involved in iron acquisition in Gc are TbpA/TbpB for human transferrin, LbpA/LbpB for human lactoferrin, and HpuA/HpuB for human hemoglobin. The ABC transport system FbpABC shuttles Fe^3+^ from the periplasm into the cytoplasm (53). While Gc does not make siderophores, the outer membrane FetA transporter binds and imports catechol-type siderophores produced by commensal bacteria, and FetBCD shuttles siderophore-bound iron into the cytoplasm (54). The genes encoding these proteins and others involved in iron responsiveness are regulated by the ferric uptake regulator Fur, which in its Fe^2+^-bound form binds conserved sequences (“Fur boxes”) to repress expression of target genes when iron is abundant. When iron is limited, apo-Fur loses affinity for Fur boxes, and repression is relieved (55, 56).

Despite our knowledge of the biochemistry and regulation of these systems, questions remain about how Gc acquires iron from different sources in the context of the inflammatory response to human infection. Here, we found that NE enables growth of Gc in iron-limited conditions. Our results indicate that NE alters iron homeostasis in Gc by derepressing Fur and increasing intracellular labile iron, a novel function for NE in bacterial subversion of nutritional immunity.

## RESULTS

### NE promotes Gc growth under iron limitation

**Fig 1:**
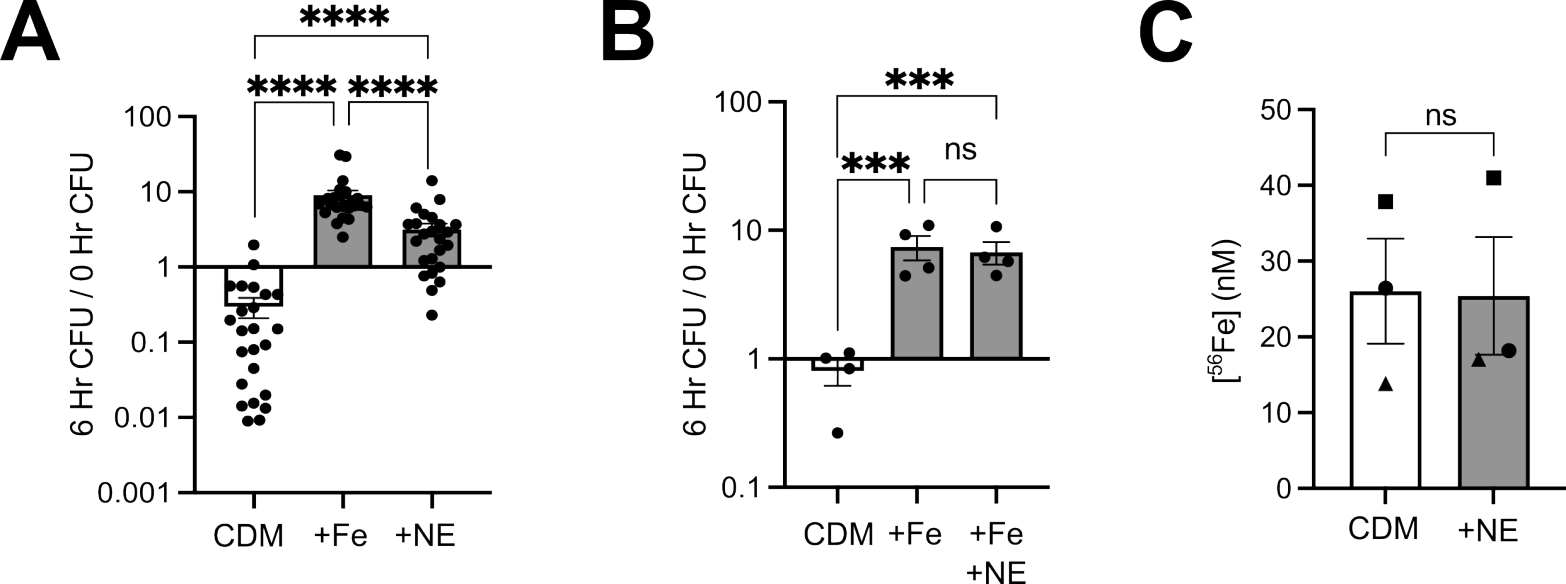
NE stimulates Gc growth in iron-limited medium. (**A**) Wild-type (WT) Gc (FA1090 background) was inoculated into Chelex-treated defined medium (CDM), CDM with 12.5 μM Fe(NO_3_)_3_ (+Fe), or CDM with 10 μM NE. (**B**) WT Gc was inoculated into CDM, CDM with 12.5 μM Fe(NO_3_)_3_ (+Fe), or CDM with 12.5 μM Fe(NO_3_)_3_ plus 10 μM NE (+Fe + NE). (**A and B**) CFUs were enumerated at 0 and 6 h by serial dilution and overnight growth on gonococcal base agar and reported as the ratio of CFU at 6 h/0 h. Significance was determined by one-way ANOVA with Tukey’s multiple comparison test on log_10_-transformed data. ns, not significant. ***P < 0.001, ****P < 0.0001. (**C**) The concentration of ^56^Fe in formulations of CDM on different days, indicated by the shapes, with or without 10 μM NE, was measured by ICP-MS. Significance was determined by paired Student’s *t*-test.

To model the metal-limited conditions that Gc experiences during infection, strain FA1090 Gc was inoculated into Chelex-treated defined medium (CDM) (57). Gc failed to grow in CDM: at 6 h, the CFUs enumerated from CDM were at or below the starting inoculum (Fig. 1A). Addition of 12.5 μM supplemental iron restored Gc growth in CDM (average of 9.1-fold increase over inoculum) (Fig. 1A; Fig. S1A). Thus, iron is the primary limiting nutrient in CDM for Gc.

To our surprise, addition of 10 µM NE was sufficient to support Gc growth in CDM, promoting on average a 3.2-fold increase in CFU over 6 h (Fig. 1A; Fig. S1A). This effect was not specific to the FA1090 wild-type (WT) strain, as NE promoted growth in MS11 and the multidrug-resistant strain H041 (WHO X) (Fig. S1B and C). However, NE did not promote growth in CDM for strains F62 or FA19 (Fig. S1D and E). Adding NE did not further increase the growth of WT Gc in CDM that contained supplemental iron, suggesting that the enhancement of growth by NE involved iron (Fig. 1B). To determine whether NE was providing contaminant iron to the medium, the concentration of ^56^Fe in CDM alone and CDM with 10 µM NE was measured by inductively coupled plasma mass spectrometry (ICP-MS). There was no significant difference in the iron content of the medium, suggesting NE was not contributing additional iron to CDM (Fig. 1C).

### NE does not act on sensor histidine kinases BasS and MisS or as a pseudosiderophore

**Fig 2:**
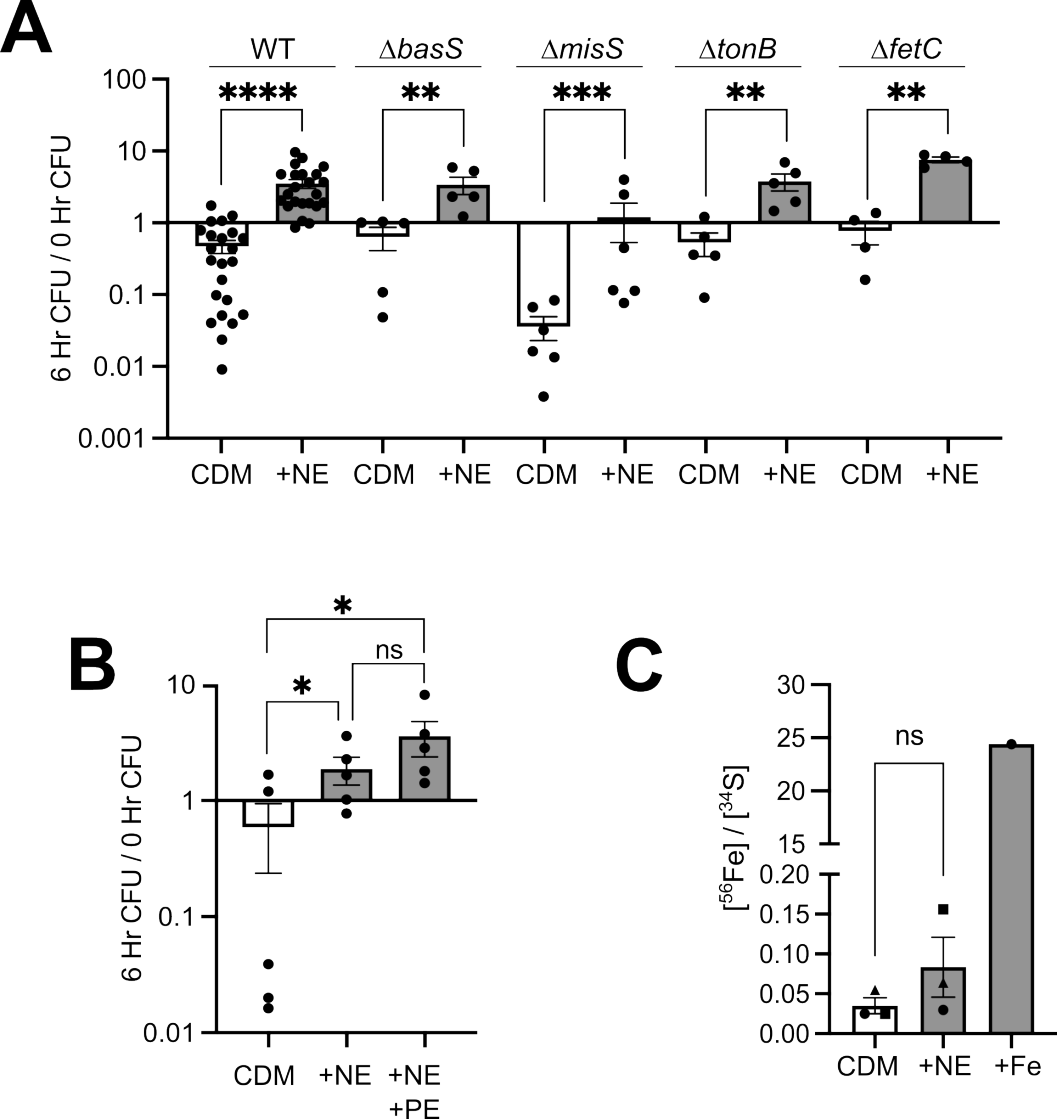
NE promotes Gc growth independent of histidine kinase sensors BasS and MisS, TonB, or the inner membrane siderophore transporter FetC. (**A**) WT Gc (FA1090 background) or the indicated mutants were inoculated into CDM with and without 10 μM NE. (**B**) WT Gc was inoculated into CDM, CDM with 10 μM NE, or CDM with 10 μM NE and 100 μM phentolamine (+NE + PE). In panels A and B, CFUs were enumerated after 0 and 6 h as in Fig. 1. Data points represent biological replicates. Asterisks represent *P* values from one-way ANOVA with Tukey’s multiple comparison test on log_10_-transformed data. *P < 0.05, ** *P* < 0.01, *** *P* < 0.001, **** *P* < 0.0001. (**C**) Gc was grown for 1 h in CDM alone, CDM with 10 μM NE, or CDM with 25 μM Fe(NO_3_)_3_ (+Fe). Bacteria were pelleted and digested, and metal concentrations were measured by ICP-MS and normalized to sulfur. Significance was determined by an unpaired Student’s *t*-test. ns, not significant.

To determine how NE stimulated iron-limited growth of Gc, we first tested the hypothesis that NE signaled through a Gc sensor histidine kinase. While Gc does not have a clear QseC homolog (58), NCBI-BLAST identified two Gc ORFs with homology to QseC from EHEC O157:H7, specifically in the C-terminal kinase domain: BasS (*ngo0112*) (32% identity) and MisS (*ngo0176*) (28% identity). Insertion-deletion mutants in each Gc gene were generated and compared to the WT parent for iron- and NE-dependent growth. NE induced growth in both the *∆basS* and *∆misS* mutants (Fig. 2A), indicating that BasS and MisS are not NE’s sole target in the gonococcus. Although phentolamine blocks QseC-mediated catecholamine sensing in other bacteria, it had no effect on the growth of Gc in CDM containing NE (Fig. 2B). Together, these data suggest that the Gc response to NE is distinct from the QseC model.

Second, we assessed the ability of NE to act as a pseudosiderophore, reasoning that Gc could use its Fet siderophore-acquisition system to scavenge and shuttle NE that is complexed to the trace iron present in CDM (~25 nM; see Fig. 1C). We tested this hypothesis by using insertion-deletion mutants in *tonB*, which powers all TonB-dependent transporters, including outer membrane catecholate xenosiderophore transporter FetA, and *fetC*, the sole xenosiderophore inner membrane transporter. Growth of the *ΔtonB* and *ΔfetC* mutants was similarly stimulated with NE compared to WT (Fig. 2A). Additionally, Gc was grown in CDM with and without NE, and bacterial metal concentrations were measured by ICP-MS, normalized to sulfur content. Compared to Gc grown in CDM alone, Gc grown in CDM with NE did not contain significantly increased total iron (Fig. 2C). From these data, we conclude that NE is not improving Gc growth by acting as a pseudosiderophore.

### NE derepresses the Fur regulon in iron-limited Gc

**Fig 3:**
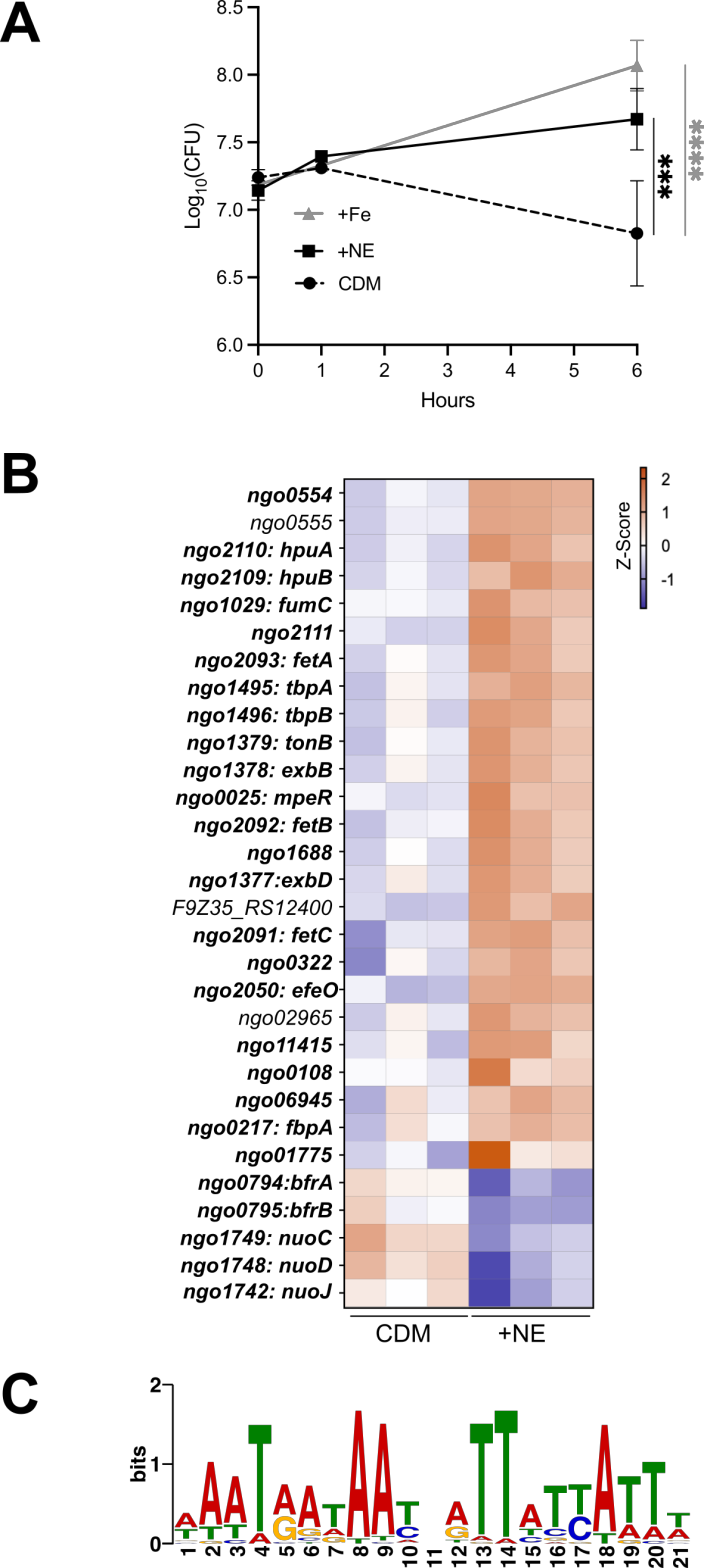
NE derepresses the Fur regulon in iron-limited Gc. WT Gc (FA1090 background) was inoculated into CDM alone, with 12.5 μM Fe(NO_3_)_3_ (+Fe), or with 10 μM NE. (**A**) CFU were enumerated at 0, 1, and 6 h. Data points represent the mean of three biological replicates, and error bars represent the SEM. Significance was determined by two-way ANOVA with Tukey’s multiple comparison test. ***P < 0.001, ****P < 0.0001. (**B**) After 1 h, bacteria were collected; RNA was extracted; and RNAseq was conducted. Thirty genes were differentially expressed with NE treatment, defined by a |Log_2_(FoldChange)| > 0.5 and an adjusted *P* < 0.05. The 25 increased differentially expressed genes are listed in order of most significant *P* value, followed by the 5 decreased differentially expressed genes, also listed in order of most significant *P* value. Colors indicate *z*-score normalized expression values. Bold genes contain a known or putative Fur box in their promoters. (**C**) The 30 differentially expressed ORFs and 500 bp upstream regions, corresponding to promoter regions, were analyzed using MEME to identify overrepresented palindromic sequence motifs *de novo*. A WebLogo of the motif derived from these data (*E* value = 4.5 × 10^−10^) is displayed.

Microbial pathogens have been reported to exploit catecholamines during infection in two ways, but our results do not support either of them for Gc. To begin to understand how NE impacts Gc physiology to promote survival under iron limitation, we took a transcriptomics approach. RNA was extracted and sequenced from triplicate cultures of Gc incubated for 1 h in CDM, with or without 10 μM NE. This timepoint was chosen because it preceded any observed growth differences between conditions (Fig. 3A). Gc was also grown with 12.5 μM Fe(NO_3_)_3_, which was sufficient to promote Gc growth in CDM (Fig. 3A). However, this concentration of iron was insufficient to repress the Fur regulon and was not analyzed further (Fig. 3A; Data set S1 and S2).

Using an FDR cutoff of 0.05 and an absolute Log_₂_(FoldChange) cutoff of 0.5, we identified 30 genes that were differentially expressed in NE-treated compared to untreated bacteria (Fig. 3B; Data set S2). Twenty-five genes were increased with NE, and five genes were decreased (Fig. 3B). Uncharacterized ORFs were assigned putative functions based on GenBank annotations (GCA_009757095.1, ASM975709v1). Differentially expressed genes broadly fell into four categories: iron acquisition and regulation, iron storage, surface lipoprotein assembly modifiers (SLAMs) and their cargo, and metabolism.

- Iron acquisition and regulation: NE-treated Gc had increased expression of genes for iron acquisition through the outer membrane, including the TonB complex, TdTs for acquisition of iron from transferrin (TbpA), hemoglobin/haptoglobin (HpuB), and xenosiderophores (FetA), and two uncharacterized putative TdTs encoded by *ngo02965* and *ngo06945*. Notably, the genes for TdTs that transport zinc (*tdfH* and *tdfJ*) were not differentially expressed, emphasizing the effect of NE on iron-dependent growth in Gc. Also upregulated were genes encoding periplasmic iron shuttles for Fe^3+^ (FbpA) and catecholate siderophores (FetB), the inner membrane xenosiderophore permease FetC, and putative periplasmic iron transporter *efeO* (*ngo2050*). *mpeR*, which encodes a regulator that activates *fetA* expression (59), was also upregulated, as was a putative AraC-type regulator encoded by *ngo11415*.
- Iron storage: The genes encoding the only bacterioferritin proteins annotated in the Gc genome, *bfrA* and *bfrB*, were significantly decreased in Gc grown with NE. *ngo01775* is predicted to encode a bacterioferritin-associated ferredoxin and was upregulated with NE, suggesting that NE may trigger iron to be released from storage.
- SLAMs: Three of four predicted SLAMs in the Gc genome were upregulated by NE (*ngo1688*, *ngo2111*, and *ngo0555*). These SLAMs facilitate lipoprotein surface exposure and possibly secrete soluble cargo (60). Transcripts encoding SLAM cargo (*tbpB*, *hpuA*, *ngo0554*) were also upregulated with NE. Fur-regulated *ngo0322*, predicted to encode a lipoprotein, was also upregulated. It is not known whether it requires a SLAM for surface expression.
- Metabolism: While NE did not induce a broad metabolic rewiring of Gc, five metabolic genes were differentially expressed. Three subunits of the NADH dehydrogenase (Complex I) of the electron transport chain—*nuoC*, *nuoD*, and *nuoJ*—were downregulated with NE. This complex relies on multiple iron-sulfur clusters as cofactors to transport electrons. *fumC*, encoding TCA cycle enzyme fumarate hydratase, and predicted FMN reductase *ngo0108* were both upregulated with NE.

Notably, 24/30 (80%) differentially expressed genes belong to operons that previously were shown to be regulated by the *Neisseria* transcription factor Fur, based on transcriptomic analyses of a *fur* mutant and/or experimentally validated Fur boxes (Table S1) (55, 56, 61–66). Gc expressed Fur-repressed genes in iron-limited CDM. However, their transcripts were significantly more abundant in Gc treated with NE (Fig. 3B). The six differentially expressed genes that have not previously been reported to be in the *Neisseria* Fur regulon include *ngo0555* and five ORFs (*ngo01775*, *ngo02965*, *ngo06945*, *ngo11415*, and F9Z35_RS12400) that are missing from earlier genome annotations. When sequences (ORF + 500 bp upstream) of all 30 differentially expressed genes were analyzed using MEME, one of the most significantly overrepresented palindromic motifs (Fig. 3C) (*E* value = 4.5 × 10^−10^) closely matched predicted Fur-binding consensus sequences (“Fur box”) previously reported for *Neisseria* spp. (62, 66, 67). This motif was ≤200 bp upstream of the translational start site of 17 differentially expressed genes/operons (Table S2) and corresponded to canonical Fur boxes that were previously defined 5′ of the translational start of *tbpB* and *fbpA* (56, 61, 64, 68, 69). Fur boxes were also identified in three of the previously unannotated genes (*ngo11415*, *ngo01775*, and *ngo06945*) (Table S2), bringing the total number of differentially expressed genes with Fur boxes to 27/30. The three differentially expressed genes lacking a Fur box were *ngo0555*, *ngo02965*, and F9Z35_RS12400. We conclude that NE enhances derepression of the Fur regulon when iron is limiting.

### NE partially alleviates Fur-mediated repression in iron-limited Gc

To test if NE works through Fur to promote the growth of Gc in iron-limiting conditions, we employed a Fur point mutation, *fur-1*, that is reported to have reduced binding to Fur target sequences (63). As expected, Gc with the *fur-1* mutation had a small colony morphology on iron-rich gonococcal base (GCB) plates. Also, when compared with WT Gc by Western blot, the *fur-1* mutant produced higher amounts of TbpB in CDM, and TbpB was not repressed by adding excess iron (Fig. S2). These phenotypes are consistent with the reported behavior of the *fur-1* mutant (63, 70).

**Fig 4:**
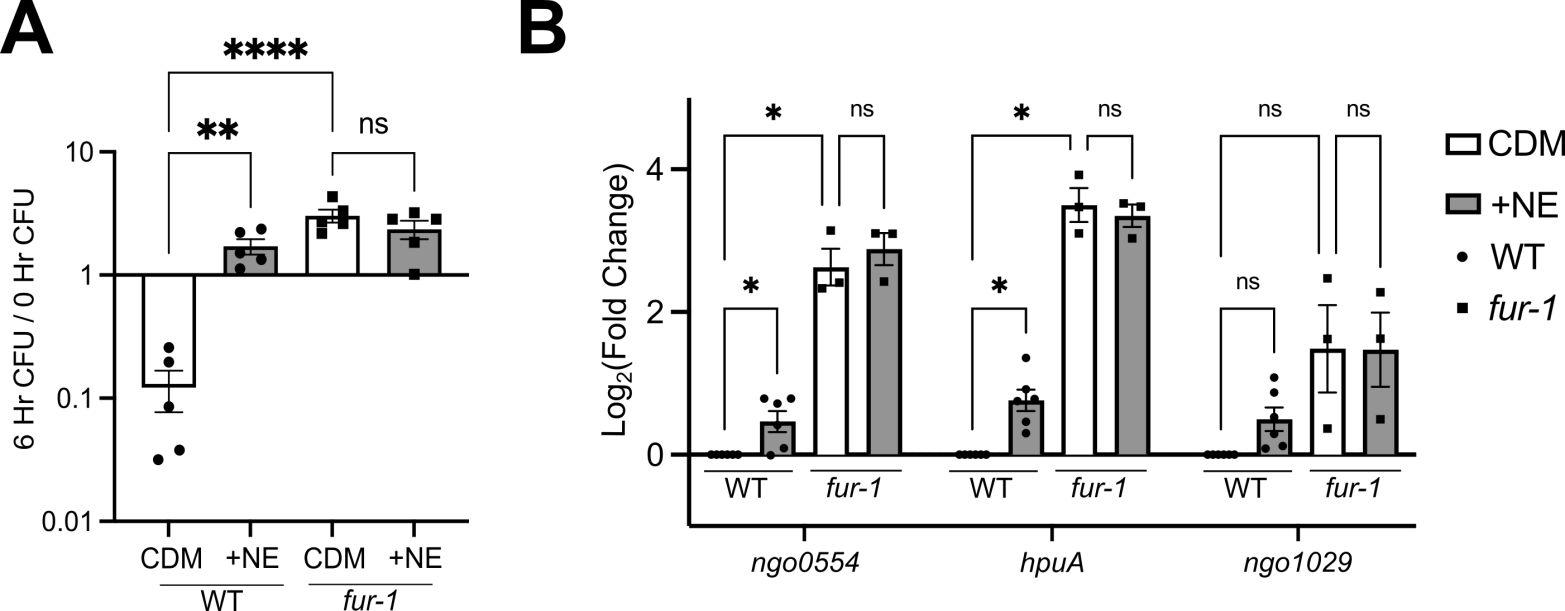
The Gc response to NE works through Fur. (**A**) WT Gc (FA1090 background) or the *fur-1* mutant was inoculated into CDM with and without 10 μM NE. CFUs were enumerated at 0 and 6 h with serial dilutions as in Fig. 1. Significance was determined by one-way ANOVA with Tukey’s multiple comparison test on log_10_-transformed data. ns, not significant. **P < 0.01, ****P < 0.0001. (**B**) RNA was extracted from WT and *fur-1* Gc grown for 1 h in CDM alone or CDM + 10 μM NE, and transcripts of interest were quantified via qRT-PCR. Fold Change was calculated as 2^−ΔΔCT^, with 5S rRNA as the internal control gene and WT Gc grown in CDM alone as the calibrator condition. Significance was determined by two-way RM ANOVA with Holm-Šídák’s multiple comparison test. *P < 0.05.

WT Gc and the *fur-1* mutant were examined for their NE-dependent growth in CDM. The *fur-1* mutant grew significantly better in CDM compared to WT (Fig. 4A). Importantly, the growth of *fur-1* was not further enhanced when NE was added, whereas WT Gc was (Fig. 4A). These results suggest that the effect of NE on the iron-limited growth of Gc is mediated through Fur.

To examine how NE affected transcript levels of Fur-regulated genes, qRT-PCR was conducted on WT and *fur-1* Gc grown in CDM ± NE for 1 h. Gene targets included three of the top five differentially expressed genes from the transcriptomics data: *ngo0554*, *hpuA*, and *ngo1029*. When cultured in CDM, these transcripts were significantly more abundant in the *fur-1* mutant compared to WT Gc, as expected (Fig. 4B). In agreement with the transcriptomics results, NE increased expression of these genes in WT Gc by qRT-PCR (Fig. 4B). However, NE did not further increase abundance of these transcripts in the *fur-1* mutant (Fig. 4B). Together with the growth data, these results support the hypothesis that addition of NE relieves repression of target genes by Fur to enable Gc growth when iron is limited.

### NE directly disrupts binding of Fur to DNA containing Fur-binding sequences

Based on the findings with the *fur-1* mutant, we hypothesized that NE directly affects the binding of Gc Fur to its target sequences, leading to derepression of the Fur regulon. This hypothesis was tested using a DNA-protein interaction (DPI)-ELISA (71, 72). Here, purified Gc Fur was incubated with a biotinylated DNA amplicon target containing a known Fur-binding sequence in the promoter of TbpB (p*tbpB*) or, as a negative control, a region of the RmpM promoter that is not bound by Fur (p*rmpM*). Under aerobic benchtop conditions, apo-Fur and Fur loaded with FeSO_4_ had the same low level of binding to p*tbpB* (Fig. S3A). We anticipated this was due to Fe^2+^ oxidation in atmospheric conditions. In support of this, Fur loaded with MnCl_2_, which mimics the chemical coordination of Fe^2+^ for *in vitro* Fur-DNA binding assays (56), had significantly increased binding to p*tbpB* compared to apo-Fur. However, there was no effect of NE (final concentration ranging from 10 μM to 10 mM) on the binding of Mn^2+^-loaded Fur to p*tbpB* (Fig. S3C). Fur did not bind to negative control p*rmpM* at any Mn^2+^ concentration (Fig. S3B and D).

**Fig 5:**
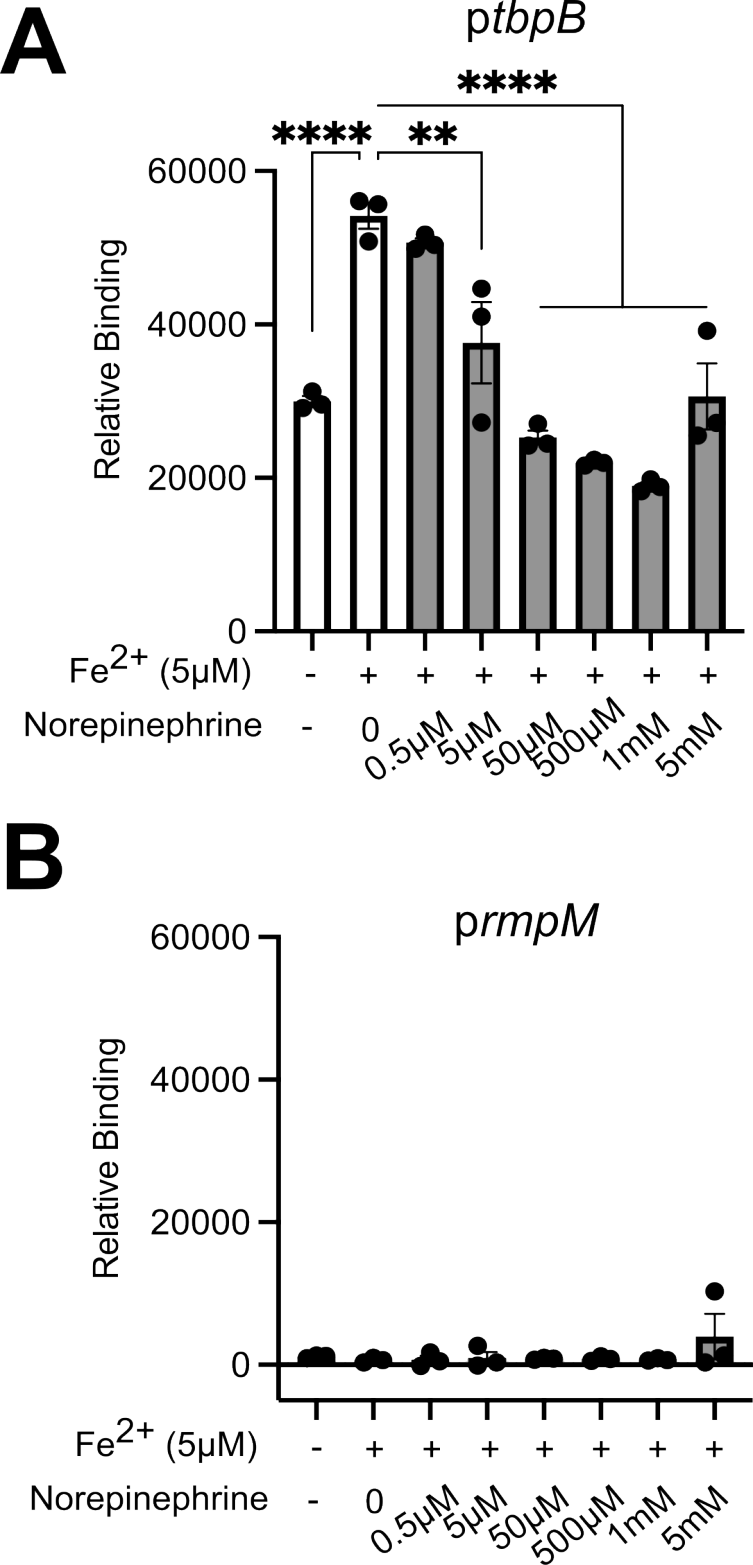
NE decreases binding of Fe^2+^-Fur to a Fur box *in vitro.* (**A and B**) His-GcFur was incubated with Fe_2_SO_4_ and indicated concentrations of NE, and binding to biotinylated promoters (p*tbpB* [**A**] or p*rmpM* [**B**]) was measured by DPI-ELISA, all under anaerobic conditions. p*tbpB* contains a fur box while p*rmpM* does not. Relative binding (relative fluorescence units) is a readout of fur binding to the indicated promoters. Significance was determined by one-way ANOVA with Tukey’s multiple comparison test. **P < 0.01, ****P < 0.0001.

In order to test how NE affected binding of Fe^2+^-Fur to its DNA target, the DPI-ELISA was conducted anaerobically to prevent Fe^2+^ from oxidizing (see Materials and Methods). NE reduced Fur binding to p*tbpB* in a concentration-dependent manner (Fig. 5A), with no effect on p*rmpM* (Fig. 5B). We conclude that NE has the ability to directly disrupt the binding of Fe^2+^-Fur to its target DNA sequences.

### NE increases Gc streptonigrin sensitivity, an indicator of labile iron

**Fig 6:**
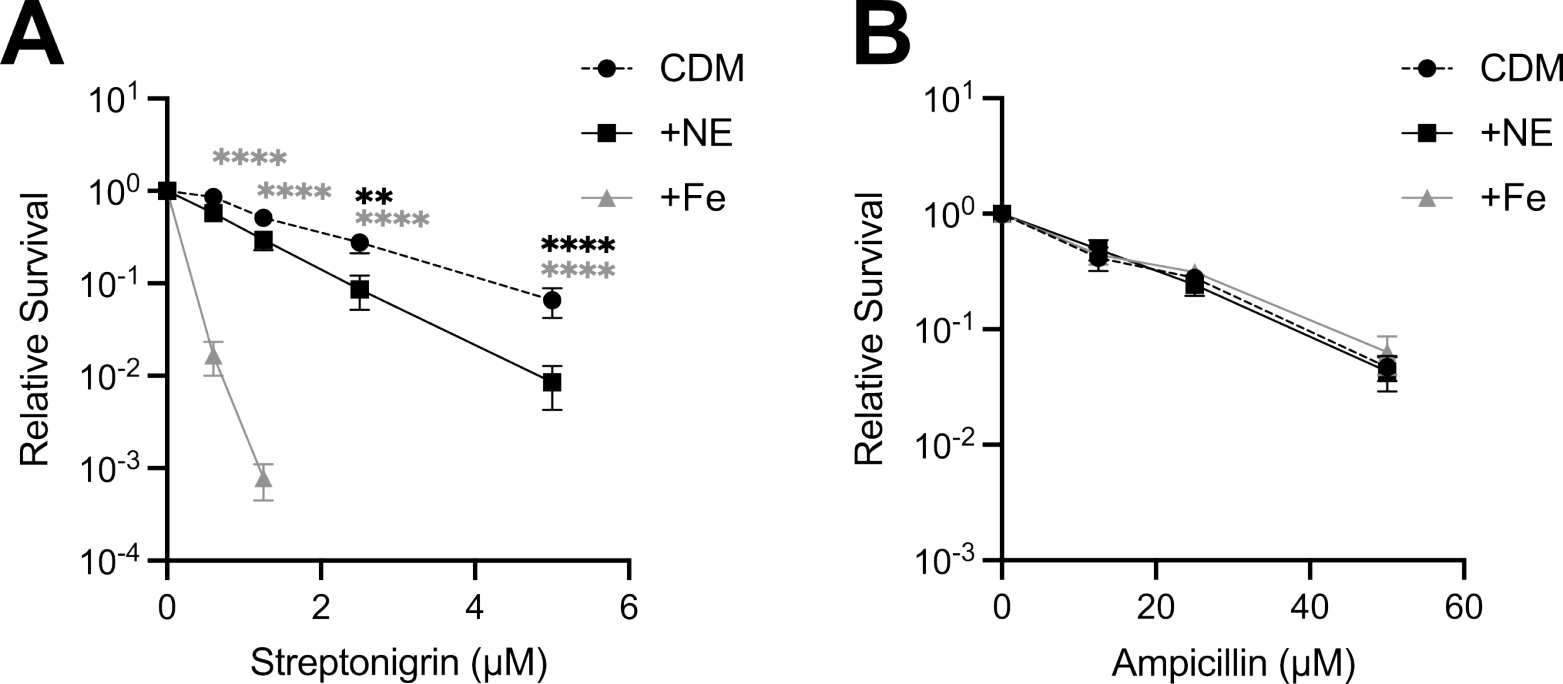
NE increases Gc streptonigrin sensitivity. WT Gc (FA1090 background) was inoculated into CDM alone, CDM with 10 μM NE, or CDM with 25 μM Fe(NO_3_)_3_ (+Fe). After 2 h, Gc was exposed to increasing concentrations of streptonigrin (**A**) or ampicillin (**B**) for 1 h. CFUs were enumerated, and data are presented as survival relative to Gc without antibiotic treatment. Data points indicate the mean of *n* = 6–9 biological replicates (**A**) or 3 biological replicates (**B**). Significance was determined by two-way ANOVA with Holm-Šídák’s multiple comparison test on log_10_-transformed data. **P < 0.01, ****P < 0.0001. Black asterisks (top) indicate significance between +NE and CDM conditions, and gray asterisks (bottom) indicate significance between +Fe and CDM conditions.

We hypothesized that NE may increase the labile iron pool in Gc, e.g., mobilizing iron from storage biomolecules. To test this, Gc was grown with 10 μM NE or 25 μM iron and then exposed to increasing concentrations of the antibiotic streptonigrin, which requires labile iron to exert activity (73). Gc grown with NE exhibited significantly increased streptonigrin sensitivity compared with the untreated control (Fig. 6A). NE did not affect sensitivity of Gc to ampicillin, whose mechanism of action is iron independent (Fig. 6B). We conclude that NE enhances Gc survival in low-iron conditions in part by increasing intracellular labile iron, enabling Gc to meet its cellular demands for iron-dependent growth.

## DISCUSSION

As a mucosal pathogen, Gc must contend with host-imposed nutritional immunity, which limits the availability of iron and other crucial transition metals. Gc overcomes nutritional immunity by expressing transporters that extract metals from metal-sequestering proteins (51, 52). Here, we made the surprising finding that multiple strains of Gc respond to the host-derived catecholamine NE, which is present at the mucosal sites Gc infects (74–76), to survive iron limitation. Specifically, NE enabled Gc growth in CDM, a protein-free medium that Gc otherwise cannot grow in due to insufficient iron availability. Our findings suggest a novel strategy by which Gc uses this host-derived factor to overcome iron limitation, consistent with the intricate interplay between Gc and its obligate human host.

There are two ways in which pathogens have been reported to use catecholamines for enhanced growth and virulence. First, some pathogens sense catecholamines with inner membrane sensor kinases that are part of two-component systems, QseC and QseE and their homologs (25–34). There are only three sensor histidine kinases described in Gc: MisS, NtrY, and NarQ (77–80). We identified a fourth gene, *basS* (*ngo0112*), by homology to the transmembrane domain of QseC. However, Gc does not encode a true QseC homolog (58). The effects of NE on Gc growth did not require gonococcal ORFs with the highest homology to QseC: *basS* and *misS*. Of the other two histidine kinases, NarQ is constitutively active and not an environmental sensor (80, 81), and the regulon of the nitrogen-responsive NtrX, the cognate response regulator to NtrY, differs from the transcriptional changes seen with NE (79). Moreover, phentolamine, which blocks QseC NE sensing in other bacteria (25, 33–36), did not block the growth effect of NE on Gc. While we have not ruled out a possible role for uncharacterized sensors in Gc, taken together, these results imply that Gc responds to NE in a manner distinct from the QseC model. Second, catecholamines have been shown to act as pseudosiderophores to cause the release of bound iron from iron-saturated nutritional immunity proteins (38). Bacteria that use NE as a pseudosiderophore do so in a TonB-dependent manner (40, 43–45). Since CDM is a protein-free medium, we hypothesized that NE was instead scavenging the (~25 nM) trace iron in CDM to deliver to Gc. However, our results do not support this hypothesis since NE-stimulated Gc growth was independent of both TonB and inner membrane catechol-type xenosiderophore transporter FetC, and there was no significant increase in intracellular iron with NE addition. FA1090 Gc is reported to have a TonB-independent, FbpABC-dependent siderophore uptake mechanism (59, 82), and it is possible that NE is taken up through this pathway. However, our transcriptomics data show a Gc response to NE that directly opposes the Gc response to supplemental iron. Together, these observations suggest that NE does not support Gc growth by directly translocating iron into the cell. Thus, the effect of NE on Gc is unique and distinct from the prevailing models in the field.

Upon NE treatment, thirty gonococcal genes were differentially expressed, which overlapped substantially with the anaerobic (9/30) (83), hydrogen peroxide (15/30) (84), iron (21/30) (56, 59, 68, 69), and Fur (24/30) (55, 56, 61–66) regulons. Under experimental conditions, WT Gc grown in iron-limited CDM did not have full derepression of its Fur targets. We anticipate this is reflective of the nutrient conditions Gc experiences in its human host, where free iron is scarce, but iron bound to nutritional immunity proteins like transferrin can be acquired by Gc via its TonB-dependent transporters (85). NE has been reported to both increase (86–89) and decrease (90–92) expression of iron-acquisition and utilization genes, including TonB and TdTs, in other gram-negative pathogens. In Gc, NE increased expression of these genes. Neither TonB nor the Fet system was necessary for the NE-dependent increase in Gc growth in CDM; however, they may be important *in vivo*, where the substrates for these systems are available. While not statistically significant, a twofold increase in iron in Gc treated with NE was measured by ICP-MS (Fig. 2C); there may be an increase in cellular iron, which would be consistent with the upregulation of iron acquisition systems observed by RNAseq. NE treatment downregulated all 14 genes of the *nuo* operon, encoding the NADH dehydrogenase (Complex I) of the electron transport chain, with *nuoC*, *nuoD*, and *nuoJ* reaching statistical and differential expression cutoffs. Since NADH dehydrogenase contains iron-sulfur clusters, reducing its abundance may liberate iron for other cellular uses. Similarly, NE-induced *fumC* and putative NADPH-dependent FMN reductase *ngo0108* encode enzymes that have been implicated in bacterial adaptation to iron limitation, representing a shift toward iron-independent metabolic pathways (93, 94). NE also downregulated genes encoding the only bacterioferritin proteins annotated in the Gc genome, *bfrA* and *bfrB*. Overall, the Gc response to NE reflects a rewiring of gene expression to increase capacity for iron uptake while enabling increased intracellular iron availability.

In *E. coli*, the iron- and Fur-regulated small RNA RyhB coordinates an iron-sparing response by promoting the degradation of mRNAs that code for the NADH dehydrogenase complex and bacterioferritin, along with other iron storage and nonessential iron-containing proteins, when iron is limited (95, 96). In Gc, NrrF has been characterized as a functional RyhB analog that alters the stability of 12 mRNAs that code for iron-using proteins (97). However, NrrF-controlled genes do not include *bfr* or *nuo*, and they do not overlap with the 30 genes that were differentially expressed with norepinephrine. There are 14 other iron-regulated sRNAs reported in Gc (67), and we have not ruled out the possibility that the effects of NE are mediated post-transcriptionally through one or multiple sRNAs.

Of the NE-induced genes, 13/25 were previously identified as Fur-repressed using a *fur* mutant in the F62 Gc strain background; all five genes that were decreased with NE were shown to be Fur activated (56, 66). The Fur regulon has not been described for strain FA1090, but it is possible that all the genes that were differentially expressed with NE are Fur-regulated in this strain, especially considering that Fur boxes were identified upstream of 27/30 of the differentially expressed genes. Twenty-four of these genes belong to operons that previously were shown to contain Fur boxes in their promoters (Table S1) (55, 56, 61–66). Fur boxes were also identified in three additional genes (putative AraC-type regulator *ngo11415*, putative bacterioferritin-associated ferredoxin *ngo01775*, and putative TdT *ngo06945*), representing new candidate targets of Fur. The most highly differentially expressed gene with NE, *ngo0554*, contains two Fur boxes within 200 bp of the translational start site, further supporting our conclusion that NE derepresses the Fur regulon. This led us to assess the growth of a *fur-1* point mutant with impaired Fur regulatory activity (63). This mutant—unlike WT—grew in CDM, and NE did not provide any additional growth benefit. Expression of NE-induced targets *ngo0554* and *hpuA* was significantly increased in the *fur-1* mutant grown in CDM and unaffected by addition of NE, showing that NE acts upstream of Fur in the response of Gc to low iron.

We uncovered two ways in which NE works via Fur to enable Gc growth in low iron conditions. First, NE can directly decrease Fur binding to target DNA sequences. Catecholamines are capable of both reducing ferric iron (37) and oxidizing ferrous iron (98, 99) due to their redox-active catechol moiety. Fur has a much lower affinity for ferric iron (Fe^3+^), and thus a lower affinity for its target DNA sequences (100). Because ferrous iron (Fe^2+^) is rapidly oxidized under normal atmospheric conditions, most studies have used Mn^2+^ as a surrogate divalent cation in Fur-binding experiments (56, 101–104). By DPI-ELISA, NE prevented Fe^2+^-Fur, but not Mn^2+^-Fur, from binding the *tbpB* Fur box promoter. The selective effect of NE on Fe^2+^-Fur versus Mn^2+^-Fur implies a redox- or chelation-driven mechanism of inactivation at the regulatory metal-binding site, rather than an effect at an allosteric site (101). Thus, our results imply that NE can either directly oxidize or chelate the Fe^2+^ in Gc Fur to inactivate it. Second, NE increased the labile intracellular iron pool in Gc, evidenced by increased streptonigrin sensitivity, an approach that has been used for bacteria including Gc (73, 105–107). While streptonigrin susceptibility increased with NE, the total iron content of Gc was not significantly increased, suggesting that NE leads to the redistribution of iron within the cell. We speculate that NE mobilizes stored iron from biomolecules like bacterioferritin, which was downregulated with NE by RNA-seq (*bfrA* and *bfrB*). Conversely, *ngo01775*, which is predicted to encode a bacterioferritin-associated ferredoxin, was upregulated with NE. Inhibition of iron mobilization from bacterioferritin has been shown to starve and kill highly virulent, multidrug-resistant strains of *Acinetobacter baumannii* and *P. aeruginosa* (108). Precisely how and where iron is redistributed within the gonococcus when iron is limited, and how NE affects it, warrant future investigation.

Of note, NE induced iron-limited growth of gonococcal strains FA1090, MS11, and H041 but did not induce growth of strains F62 or FA19 under the conditions tested. This discrepancy could be explained by strain-specific differences in iron homeostasis and regulation, including iron-regulated sRNAs or transcriptional regulators like *fur* (56, 67), as F62 is the only strain for which a full *fur* knockout has been successfully generated (66). The strain specificity of NE may also be linked to strain-specific differences in Gc iron acquisition (82): for instance, FA1090 has both TonB-dependent and TonB-independent mechanisms for catecholate siderophore uptake, while FA19 uses a strictly TonB-independent mechanism (59). Future dissection of iron homeostasis, regulation, and acquisition pathways may reveal the basis of strain-specific differences in responding to NE.

How Gc survives iron limitation has been largely attributed to its high-affinity TdTs that strip iron from human nutritional immunity proteins (51); derepression of the Fur regulon results in upregulation of TdTs that import iron (56). Here, we uncovered a new way in which Gc responds to trace iron, involving the host-derived catecholamine NE. Addition of NE enables Gc growth under trace iron conditions that is independent of TonB in an experimental system lacking all host proteins. These results highlight the importance of the iron and Fur regulon in maintaining iron homeostasis in Gc, beyond the induction of TonB-dependent iron acquisition. NE is produced at mucosal surfaces as well as by immune cells, both of which are players in Gc pathogenesis; future studies will examine how Gc responds to NE in the context of mucosal infection and inflammation. By demonstrating that the host hormone NE derepresses the Fur regulon to alter bacterial iron homeostasis and increase Gc survival, the work presented here provides a novel mechanism for how Gc survives iron limitation within its obligate human host. Refining our understanding of this process may inform the development of more effective treatments for drug-resistant gonorrhea.

## MATERIALS AND METHODS

### Gonococcal strains and mutant construction

The WT strain used for these studies is a FA1090 derivative that constitutively expresses OpaD and no other Opa proteins, and is ∆*lbpA* and *hpuAB* phase-off (109). *∆tonB*, *∆fetC*, *∆basS*, and ∆*misS* mutants were generated by spot transforming WT Gc with genomic DNA from relevant strains (Table 1). ∆*tonB* Gc was selected on spectinomycin (80 μg/mL) GCB plates and confirmed by PCR using the primers in Table 2. *∆fetC*, *∆basS*, and *∆misS* Gc were selected on kanamycin (50 μg/mL) GCB and confirmed by PCR using the primers in Table 2.

**TABLE 1:** Gc strains used in this study

Strain	Parent	Transformed with	Source
OpaD (WT)	FA1090		(109)
*∆tonB*	OpaD	MCV656 FA1090 *tonB::Ω*	(110)
*∆fetC*	OpaD	FA1090 *fetC::kan*	Hank Seifert, ordered gene knockout library for *Neisseria gonorrhoeae*
*∆basS*	OpaD	FA1090 *basS::kan*	Hank Seifert, ordered gene knockout library for *Neisseria gonorrhoeae*
*∆misS*	OpaD	JK102 FA19 *misS::kan*	William Shafer (78)
*fur-1*	OpaD	AKK548 MS11 *fur-1*	Joseph Dillard (70) (originally generated by Thomas and Sparling [63])
MS11			Joseph Dillard
H041 (WHO X)			Ann Jerse
F62			Ann Jerse
FA19			William Shafer

**TABLE 2:** Primers used in this study

Primer name	Sequence	Application
NGO0112 F	CAGGTCAGAATCAGCCTTGCC	∆*basS* transformant confirmation
NGO0112 R	GATGTAAAAAATGCCGTCTGAAGCC	∆*basS* transformant confirmation
NGO0176/0177 F	GAGTAATACGCGGCTCATGG	∆*misS* transformant confirmation
NGO0176/0177 R	GCGGACGGTATGTGCGTGA	∆*misS* transformant confirmation
NGO2091 F	GGTATTGTTTGCCGTCAGCC	∆*fetC* transformant confirmation
NGO2091 R	CATGCGTCCGATAATGTCGC	∆*fetC* transformant confirmation
NGO1379 F	GCCTATGAATGGAGCAGGC	∆*tonB* transformant confirmation
NGO1379 R	CAGGACGGGATCGCCCG	∆*tonB* transformant confirmation
fur_up F	CGGTGTCATGTGTGTTCC	*fur* forward for amplifying MS11 *fur-1* point mutation
fur_down R	TACGCATGGCAGTTCTCC	*fur* reverse for amplifying MS11 *fur-1* point mutation
NGO0554 F	GGTGAAATTACAGCCACATTCG	qRT-PCR: *ngo0554*
NGO0554 R	CAACACCGTTAAAGCTCAATTCC	qRT-PCR: *ngo0554*
NGO2110 F	AGGAGTAGCTGACGGTTATGG	qRT-PCR: *hpuA*
NGO2110 R	ACATCCGGCATTTCCATTCG	qRT-PCR: *hpuA*
NGO1029 F	ATCAACCGCCACCTTATTCC	qRT-PCR: *ngo1029*
NGO1029 R	CATCCTGCAAGTGGGTACG	qRT-PCR: *ngo1029*
5S rRNA F	CGGCCATAGCGAGTTGGT	qRT-PCR: 5S rRNA
5S rRNA R	TTGGCAGTGACCTACTTTCG	qRT-PCR: 5S rRNA
oGSU303	TTGCCGCAACTTGGAAAGTGC	Amplifying promoter with Fur box (P*_tbpB_*)
oGSU304	/5BiotinTEG/CAAGATCGAAACTGCCGCCT	Amplifying promoter with Fur box (P*_tbpB_*)
oGSU385	CAACGGCAATCGTGCGATATGG	Amplifying promoter without Fur box (P_rmpM_)
oGSU386	/5BiotinTEG/GAAGCGAGCAATGCAACGAA	Amplifying promoter without Fur box (P_rmpM_)

The *fur-1* mutant was generated using the methods described in reference 70. The *fur-1* allele was PCR amplified with fur_up-F and fur_down-R (Table 2) primers using gDNA from strain AKK548 as a template. The PCR product was resolved by gel electrophoresis, and the desired product was excised and purified using the QIAquick Gel Extraction Kit (Qiagen) following the manufacturer’s protocol and used to transform Gc on GCB plates. Transformants were identified by their small colony size and confirmed by sequencing the *fur* gene. As in reference 70, this mutant contains a Y82C substitution, an E48G substitution, and a silent ATT-to-ATC base change within isoleucine 7.

### Gc growth conditions

Gc was prepared for experiments by inoculating −80°C stocks onto GCB medium (Difco) containing Kellogg’s supplements I (111) and 1.25 μM Fe(NO_3_)_3._ Gc was cultured at 37°C with 5% CO_2_ for 16 h. Gc colonies from GCB plates were inoculated to a final optical density at 600 nm (OD_600_) of 0.05 in 5mL of defined medium that was pretreated with Chelex-100 resin (Bio-Rad) (Chelex-treated defined medium [CDM]) (57) in metal-free 15 mL conical tubes (VWR 89049-170). Cultures were grown at 37°C for 2 h, during which they doubled to an OD of ≥0.10 and were then used in experiments.

### CDM growth assays

Bacteria were cultured as above, diluted to an OD of 0.02 in CDM, and 20 μL of bacterial suspension was inoculated into 180 μL of indicated medium (starting concentration = 2 × 10^6^ CFU/mL). Where indicated, Fe(NO_3_)_3_ was used as an iron source. Catecholamine stocks (10 mM) in CDM were prepared fresh immediately before use. At indicated timepoints, serial dilutions were performed in GCB liquid and plated on GCB plates. After 20 h, colonies were enumerated, and CFU per milliliter was calculated. Results are presented as the ratio of 6-h CFU to 0-h CFU.

### ICP-MS

For media iron content, CDM alone, with Fe(NO_3_)_3_ or with NE, was acidified to a final concentration of 2% nitric acid (ICP grade, Thermo Scientific T00309) and analyzed by ICP-MS. For Gc iron content, Gc (5 mL cultures, OD 0.05) were grown for 2 h in CDM from overnight lawns, and NE or iron was spiked in for one additional hour. Bacteria were pelleted and washed twice with phosphate-buffered saline (PBS). Fifty microliters of ICP-grade nitric acid was added to each sample, and samples were digested at 85°C overnight before being diluted with 2 mL of UltraPure water and analyzed by ICP-MS. Media iron content was reported as molarity, while bacterial iron content was normalized to bacterial sulfur levels to account for any differences in biomass.

### RNA extraction

Gc (2 × 10^7^ CFU/mL) was inoculated into four wells of a six-well plate containing 4 mL of CDM alone, with 12.5 µM Fe(NO_3_)_3_, or with 10 µM NE at 37°C in 5% CO_2_. After 1 h, bacteria from three replicate wells (12 mL total) were pooled together in a 15 mL conical tube and pelleted at 4,000 × *g* for 11 minutes. Gc was resuspended in 200 μL RNAprotect bacteria reagent (Qiagen 76506) and stored at −80°C until processed to purify RNA. Samples were lysed according to the “Enzymatic Lysis and Proteinase K Digestion of Bacteria” protocol from Qiagen before proceeding with the RNeasy Plus Mini Kit (Qiagen 74134) per manufacturer’s instructions. DNase treatment was completed using a TURBO DNAfree kit (Invitrogen AM1907).

### RNA sequencing

RNA sample QC, bacterial rRNA depletion, RNA-seq library preparation, and Illumina RNA sequencing were conducted by the Genomics Resource Center of the Institute of Genome Sciences at the University of Maryland. rRNA reduction was completed using NEBNext rRNA Depletion Kit (Bacteria) (NEB E7850), and RNA library prep was done using NEBNext Ultra II Directional RNA Library Prep Kit (NEB E7760). Sequencing was performed using NovaSeq S2 on the NovaSeq6000 platform, with 30 M paired-end 150 bp read pairs per sample.

Reads were checked for quality using FASTQC (112) (v.0.12.0) and trimmed using Trimmomatic (113) (v.0.39). Trimmed reads were uploaded to the Galaxy server (114), where subsequent analyses were performed. Trimmed reads were aligned to the *Neisseria gonorrhoeae* FA1090 genome with GenBank annotations (GCA_009757095.1, ASM975709v1) using Bowtie 2 (115) (v.2.5.3). Gene-level read counts were generated from aligned RNA-seq reads using HTSeq (116) (v.2.0.5). Significant genes were called using DESeq2 (117) (v.2.11.40.8), using fold change cutoffs and *P* value cutoffs of 0.5 and 0.05, respectively. A heatmap was generated in GraphPad Prism using *z*-score-normalized VST counts. RNA-seq data are deposited at the Gene Expression Omnibus database under accession no. GSE312203.

### DNA motif prediction

Differentially expressed genes and their corresponding promoter regions (open reading frame plus 500 bp upstream) were analyzed using MEME (118) (v.5.5.8) on the Galaxy server (114) to identify overrepresented palindromic sequence motifs *de novo*. The analysis was performed, allowing any number of sites per sequence, with sites on either strand and a motif width of 18–21 bp. The consensus sequence was visualized as a WebLogo (119). Sites that contributed to the construction of the motif were mapped back to the FA1090 genome to calculate their distance from the associated ORF (Table S2).

### qRT-PCR

RNA was extracted as above from WT and *fur-1* Gc grown in CDM alone and CDM + 10 μM NE. Transcripts of interest were quantified via qRT-PCR using Power SYBR Green PCR Master Mix (Thermo Fisher Scientific, 4367659), RNase inhibitor (Life Technologies, N8080119), Multiscribe Reverse Transcriptase (Life Technologies, 4311235), and validated qRT-PCR primers (Table 2). Quantitation occurred on a Thermo Fisher QuantStudio 3 instrument. qRT-PCR data were analyzed using the comparative *C*_*T*_ method (120). 5S rRNA was used for an internal control gene, and WT Gc grown in CDM alone was used as the calibrator condition.

### Fur DPI-ELISA

The His-tagged gonococcal Fur protein (His-GcFur) was expressed and purified as described previously (72). Biotinylated primers were used to PCR-amplify biotinylated DNA probes. DPI-ELISA was based on protocols by Brand et al. (71) and Padmanabhan et al. (72). For assays that used Fe^2+^ as the divalent cation, the anaerobic chamber was used. Biotinylated PCR DNA, the streptavidin-coated plate, and His-GcFur were entered into the anaerobic chamber on the morning of the experiment; all other reagents were equilibrated in the anaerobic chamber for 1 week prior to use.

Biotinylated PCR DNA was diluted to 12.5 pmol in 1× low-salt tris buffered saline (LS-TBS), bound (100 μL/well) to a prewashed streptavidin-coated high-capacity plate (Pierce High-Capacity Plates, Cat 15500), and incubated for 2 h at room temperature. After 2 h, the plate was washed five times with 200 μL 1× PBS. A 5% milk blocker (in 1× LS-TBS with 0.05% Tween 20 and 0.05% sodium azide) was added at 200 μL per well and incubated for 1 h at room temperature. His-GcFur (10 μM) was loaded with 5 μM iron(II) sulfate heptahydrate or manganese(II) chloride tetrahydrate and increasing concentrations of NE. Metal-loaded His-GcFur was added to the DNA bound plate for 1 h at room temperature. Anti-His monoclonal antibody (33D10.D2.G8) coupled to DyLight 488 (Rockland 200-341-382) was diluted in a blocker at 1:1,000 and added to the plate. The plate was washed five times with PBS as above, and 200 μL of PBS was added for detection. Fluorescence (485/528 nm) was read using the Synergy 2 Plate Reader (BioTek). To determine the optimal divalent cation concentration for this assay, we used 10 μM of Fur and titrated Mn^2+^ (Fig. S3B).

### Streptonigrin and ampicillin sensitivity assays

Piliated WT Gc was grown on GCB plates overnight as described in Gc Growth Conditions. Gc colonies from plates were inoculated to a final OD600 of 0.10 in 16 mL of CDM in metal-free 50 mL conical tubes. Cultures were split into three 15 mL conical tubes (5 mL each), and NE or Fe(NO_3_)_3_ was added to a final concentration of 10 or 25 μM, respectively. Cultures were grown at 37°C for 2 h, during which they grew to an OD of ≥0.15. After 2 h, cultures were normalized to the lowest OD (cultures were typically within 0.03 OD units of each other). Streptonigrin from *Streptomyces flocculus* (Sigma-Aldrich, S1014) was resuspended in DMSO to a stock concentration of 20 mM. Ampicillin sodium salt (Sigma-Aldrich, A9518) was resuspended in 70% ethanol to a stock concentration of 100 mg/mL (270 mM). One hundred microliters of 2× indicated antibiotic concentrations in CDM were added to a 96-well plate, and 100 μL of bacterial culture was inoculated. At 0 and 1 h, cultures were plated, and CFUs were enumerated after overnight growth on GCB agar. Bacterial survival is presented relative to CFU without antibiotic.

### Statistical analyses and data representation

Statistical tests were conducted in GraphPad Prism 10.0.3. Statistical tests with appropriate post hoc tests are indicated in each figure legend. Bars represent the mean, and error bars represent the standard error of the mean. Unless otherwise specified, data points represent individual biological replicates.

## Data Availability

RNAseq data have been deposited at Gene Expression Omnibus under accession no. GSE312203.

## SUPPLEMENTAL MATERIAL

Data set S1 jb.00597-25-s0001.xls Raw and VST normalized counts.

Data set S2 jb.00597-25-s0002.xlsx Genes differentially expressed after iron or norepinephrine treatment.

Supplemental figures and tables jb.00597-25-s0003.pdf Figures S1 to S3, and Tables S1 and S2.

ASM does not own the copyrights to Supplemental Material that may be linked to, or accessed through, an article. The authors have granted ASM a non-exclusive, world-wide license to publish the Supplemental Material files. Please contact the corresponding author directly for reuse.
