# Supplementary material for: Norepinephrine derepresses the Fur regulon of Neisseria gonorrhoeae to enable growth in iron-limited conditions
Source: J Bacteriol. 2026 Jan 20;208(2):e00597-25. doi: 10.1128/jb.00597-25 (PMC12918729; doi:10.1128/jb.00597-25)
Supplement: Supplemental figures and tables — Figures S1 to S3, and Tables S1 and S2. [file jb.00597-25-s0003.pdf]

<u>Supplemental Material</u>

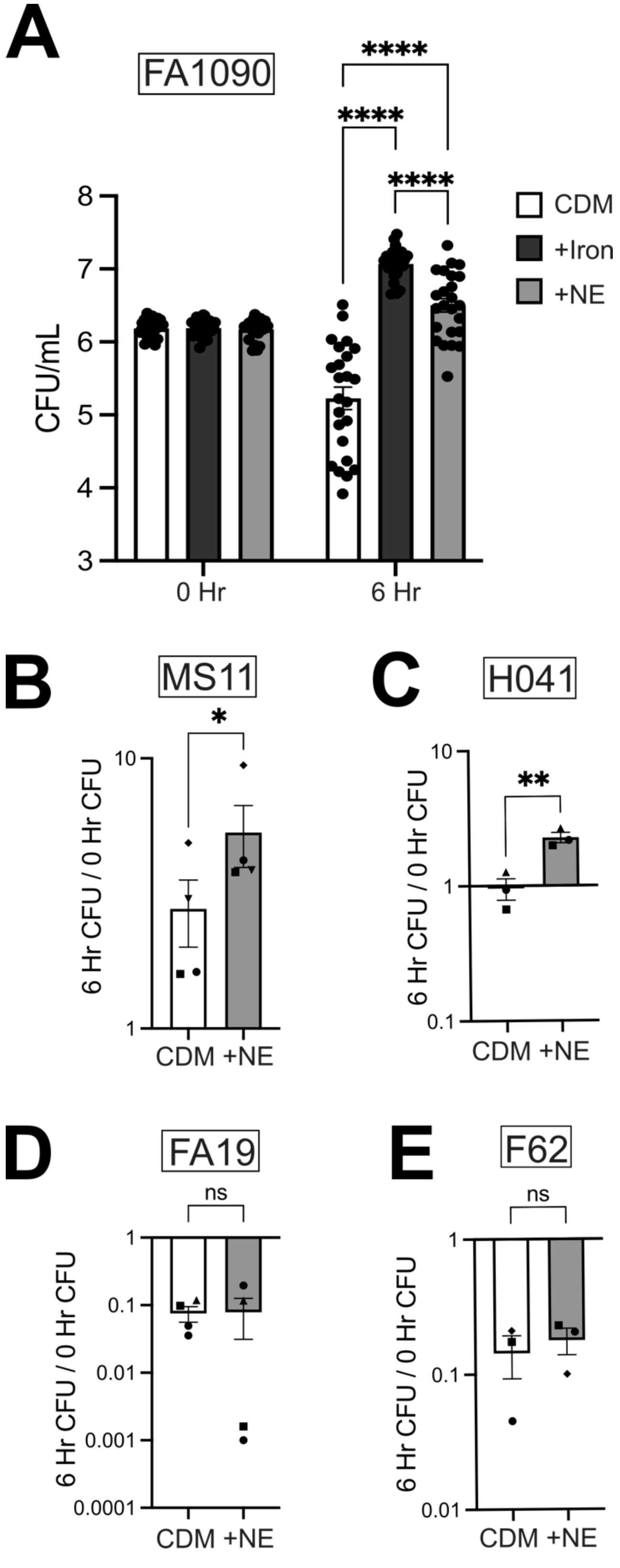

Figure S1. NE promotes growth of gonococcal strains FA1090, MS11, and H041 in iron-limited medium. The indicated strains of Gc were inoculated into Chelex-treated Defined Medium (CDM), CDM with 12.5 μM $Fe(NO_3)_3$ (+Fe), or CDM with 10 μM NE. CFUs were enumerated at 0 and 6 hours as in **Fig 1A. A)** presents the CFU/ml at 0 and 6 hours

from **Fig 1A**. Significance was determined by two-way ANOVA with Holm-Šídák's multiple comparison test, **** $p<0.0001$. **B-E)** present the ratio of CFU at 6 hr / 0 hr for the indicated Gc strains. Shapes indicate paired data from the same biological replicate. Significance was determined by paired *t*-test, ns not significant, *$p<0.05$, **$p<0.01$.

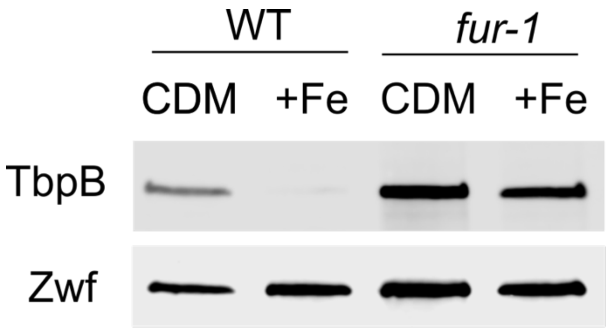

Figure S2. Derepression of TbpB production, independent of iron, in the *fur-1* mutant. WT Gc or the *fur-1* mutant was inoculated into CDM or CDM with 25 µM $Fe(NO_3)_3$ (+Fe). After 2 hours, bacteria were collected, and lysates were prepared from equal CFUs from each condition. Lysates were separated by 4-20% gradient SDS-PAGE, transferred to a nitrocellulose membrane, and stained with guinea pig anti-TbpB polyclonal antisera and rabbit anti-Zwf antisera (loading control). A representative blot from one of three independent experimental replicates is shown.

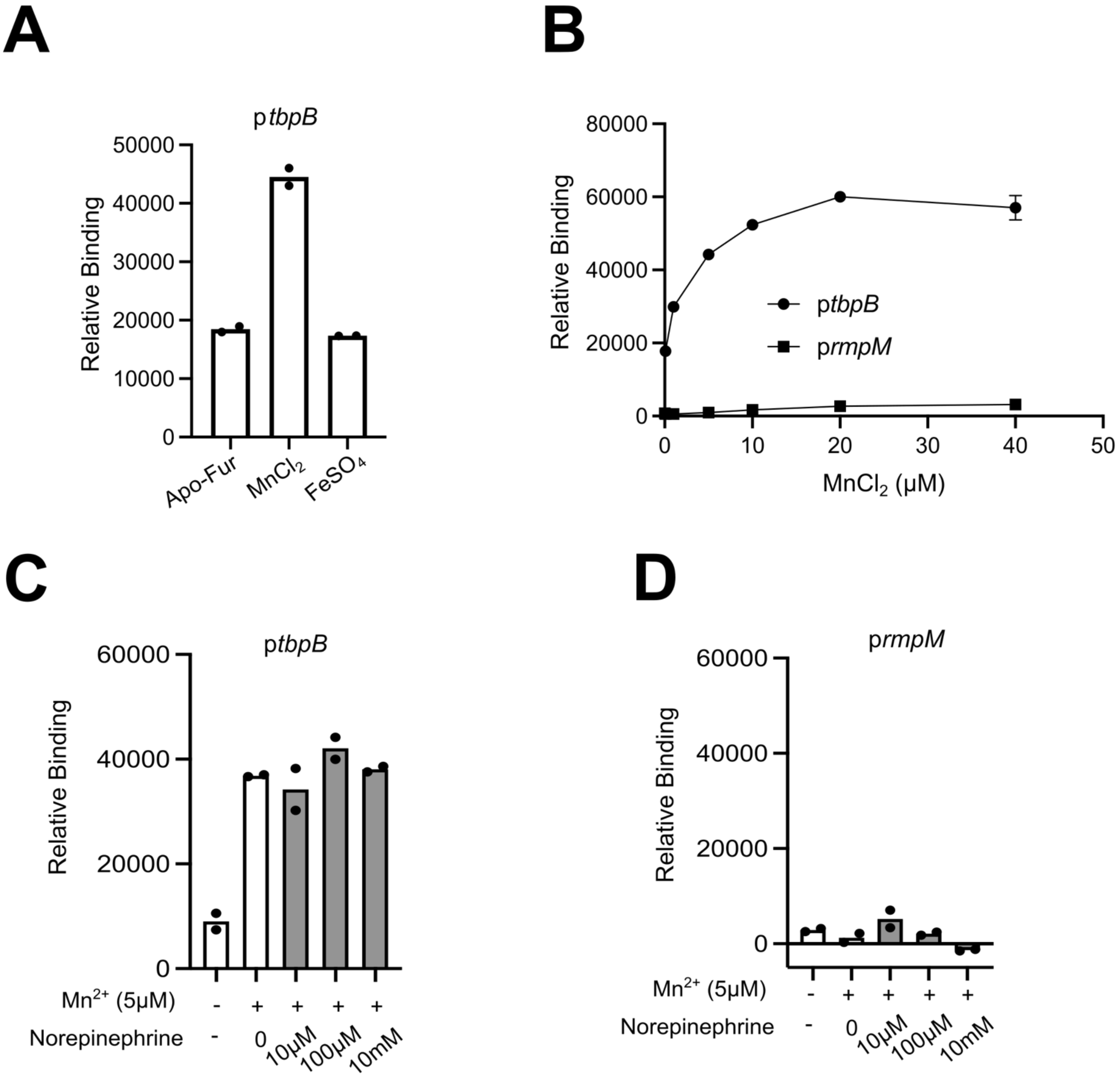

Figure S3. NE does not affect Mn^{2+}-Fur binding to Fur promoter in aerobic conditions. His-GcFur was incubated with $MnCl_2$ **(A-D)** or $FeSO_4$ **(A)** and binding to biotinylated probes (p*tbpB* **(A-C)** or p*rmpM* **(D)**) was measured by DPI-ELISA, all under aerobic conditions. Relative Binding (Relative Fluorescence Units) was measured as in **Fig. 5**.

Table S1. Regulation of DE Genes

Gene	ORF (FA1090 designation)	*in silico* Fur box?	Source *(in silico)*	Experimentally validated Fur binding?	Source (experimental validation)	Fur regulated? (WT vs Fur mutant expression)	Iron-regulated? (expression with iron exposure)
	NGO0554	yes	PMC2798271, this study	yes, FurTa	PMC2798271	repressed (PMC4966432)	repressed (PMC4966432, PMC1169496)
	NGO0555						
hpuA	NGO2110	yes	PMC2798271, PMC135177, this study	yes, FurTa	PMC2798271	repressed (PMC4966432)	repressed (PMID9157245)
hpuB	NGO2109	yes	2nd gene in an operon with *hpuA*				repressed (PMID9157245)
fumC	NGO1029	yes	PMC135177	yes, EMSA	PMC135177	repressed (PMC4966432)	repressed (PMC4966432)
	NGO2111	yes	this study			repressed (PMC4966432)	repressed (PMC4966432)
fetA	NGO2093	yes	PMC2798271, this study	yes, FurTa	PMC2798271		repressed (PMC4966432, PMC1169496, PMC3232672)
tbpA	NGO1495	yes	2nd gene in an operon with *tbpB*	yes, FurTa	PMC2798271	repressed (PMC4966432, PMC178181)	repressed (PMC4966432, PMC1169496, PMC5035231)
tbpB	NGO1496	yes	PMC2798271, this study	yes, FurTa	PMC2798271	repressed (PMC4966432, PMC178181)	repressed (PMC4966432, PMC1169496, PMC5035231)

Gene	ORF (FA1090 designation)	*in silico* Fur box?	Source (*in silico*)	Experimentally validated Fur binding?	Source (experimental validation)	Fur regulated? (WT vs Fur mutant expression)	Iron-regulated? (expression with iron exposure)
tonB	NGO1379	yes	PMC2798271, PMC135177, this study	yes, EMSA	PMC135177	repressed (PMC4966432)	repressed (PMC4966432)
exbB	NGO1378	yes	2nd gene in an operon with *tonB*	yes, FurTa	PMC2798271		repressed (PMC4966432, PMC1169496)
mpeR	NGO0025	yes	PMC2798271, this study			repressed (PMC4966432)	repressed (PMC4966432, PMC1169496)
fetB	NGO2092	yes	PMC2798271, this study	yes, FurTa/EMSA	PMC2798271	repressed (PMC4966432, PMC178181)	repressed (PMC4966432, PMC1169496, PMC3232672)
	NGO1688	yes	PMC2798271, this study			repressed (PMC4966432)	repressed
exbD	NGO1377	yes	3rd gene in an operon with *tonB*			repressed (PMC4966432)	repressed
	F9Z35_RS12400						
fetC	NGO2091	yes	in the *fet* operon (NGO2093 to NGO2088)				repressed(PMC3232672)
	NGO0322	yes	PMC2798271, this study	yes, FurTa	PMC2798271	repressed (PMC4966432)	repressed (PMC4966432, PMC1169496)
efeO	NGO2050	yes	PMC170954	yes, EMSA (*N. meningitidis* NMB0034-0036)	PMC170954	repressed (PMC170954)	
	NGO02965						
	NGO11415	yes	this study				

Gene	ORF (FA1090 designation)	*in silico* Fur box?	Source *(in silico)*	Experimentally validated Fur binding?	Source (experimental validation)	Fur regulated? (WT vs Fur mutant expression)	Iron-regulated? (expression with iron exposure)
	NGO0108	yes	PMC2798271, this study	yes, FurTa	PMC2798271		repressed (PMC1169496)
	NGO06945	yes	this study				
fbpA	NGO0217	yes	PMC2798271, this study	yes, EMSA/footprinting/FurTa	PMC178289, PMC179073, PMC2798271	repressed (PMC4966432, PMC178181)	repressed (PMC4966432)
	NGO01775	yes	this study				
bfrA	NGO0794	yes	PMC2798271, this study	yes, EMSA/footprinting/FurTa	PMC2798271, PMC3302472	induced (PMC4966432)	
bfrB	NGO0795	yes	2nd gene in an operon with *bfrA*			induced (PMC4966432)	
nuoC	NGO1749	yes	PMC2798271	yes, EMSA/footprinting/FurTa	PMC2798271, PMC3302472	induced (PMC4966432)	induced (PMC4966432)
nuoD	NGO1748	yes	PMC2798271	yes, EMSA/footprinting/FurTa	PMC2798271, PMC3302472	induced (PMC4966432)	induced (PMC4966432, PMC1169496)
nuoJ	NGO1742	yes	in the *nuo* operon (NGO1737 to NGO1751)			induced (PMC4966432)	induced (PMC4966432)

Table S2. Fur Box Predictions

Gene	ORF (FA1090 designation)	Motif(s) identified in MEME	Distance from ORF start	Contig	Start	End
	NGO0554	AATTAAAACTCATTATCAAAT	135	NZ_WHPG01000002.1	578999	579019
	NGO0554	CTATGATATTTGTATTTATTA	193	NZ_WHPG01000002.1	579077	579097
hpuA	NGO2110	TAATCAAAACTATTATTAATA	23	NZ_WHPG01000004.1	146411	146431
	NGO2111	TAATCAAAACTATTATTAATA	199	NZ_WHPG01000004.1	146411	146431
fetA	NGO2093	AGCTGATTATCGTTTTTATTT	68	NZ_WHPG01000004.1	123108	123128
tbpB	NGO1496	AAATAATAATCCTTATCATTC	22	NZ_WHPG01000007.1	137137	137157
tonB	NGO1379	TAATAGCAACAATTCCTATTT	13	NZ_WHPG01000007.1	257884	257904
mpeR	NGO0025	AAATAGGAATTATTACCAATA	35	NZ_WHPG01000004.1	226230	226250
	NGO0025	GAATAAGAATGTTTATTATTA	192	NZ_WHPG01000004.1	226073	226093
fetB	NGO2092	ATTTGATATAGATTATCATTT	73	NZ_WHPG01000004.1	120820	120840
	NGO1688	GGTTGATAATGGTTATTTTTT	overlaps start codon	NZ_WHPG01000003.1	293526	293546
	NGO0322	AAATAGGAACAATTATCATTT	70	NZ_WHPG01000004.1	526436	526456
efeO	NGO2050	AAATGCAAGTTATTTTTATCT	overlaps *efeU* start codon	NZ_WHPG01000004.1	75031	75051
	NGO11415	TTCTGATAAAGGTTATCATTT	72	NZ_WHPG01000004.1	153794	153814
	NGO0108	TAATGGTAACGATTATTATAT	39	NZ_WHPG01000004.1	326524	326544
	NGO06945	TTCTGATAATCATTAATATTT	56	NZ_WHPG01000007.1	327550	327570
fbpA	NGO0217	AAATGCAAATAATTATTTTTT	79	NZ_WHPG01000004.1	427071	427091
	NGO01775	CAATGCGAACCGTTTTTATTT	64	NZ_WHPG01000004.1	531852	531872
bfrA	NGO0794	TTTTGATAATTGTTTGTATTT	134	NZ_WHPG01000002.1	326094	326114
bfrA	NGO0794	TATAATGAATCTTTCTCATTA	312	NZ_WHPG01000002.1	326272	326292
